# Integrated Analysis of Transcriptomic and Proteomics Data Reveals the Induction Effects of Rotenoid Biosynthesis of *Mirabilis himalaica* Caused by UV-B Radiation

**DOI:** 10.3390/ijms19113324

**Published:** 2018-10-25

**Authors:** Li Gu, Weilie Zheng, Mingjie Li, Hong Quan, Jianming Wang, Fengji Wang, Wei Huang, Yunfang Wu, Xiaozhong Lan, Zhongyi Zhang

**Affiliations:** 1 Key Laboratory of Ministry of Education for Genetics, Breeding and Multiple Utilization of Crops, College of Crop Science, Fujian Agriculture and Forestry University, Fuzhou 350002, China; guli5101@163.com (L.G.); xinyuzszj@163.com (M.L.); wjm1009@126.com (J.W.); wangfengji@fafu.edu.cn (F.W.); wyf@m.fafu.edu.cn (Y.W.); 2 Medicinal Plants Research Centre, Tibet Agricultural and Animal Husbandry University, Nyingchi 860000, China; xzzhengweilie@21cn.com (W.Z.); qnhvgm@gmail.com (H.Q.); 3 College of Life Science, Fujian Agriculture and Forestry University, Fuzhou 350002, China; weiyena2001@gmail.com

**Keywords:** *Mirabilis himalaica*, UV-B radiation, plant hormone signal transduction, phosphatidylinositol signaling system, rotenoid biosynthesis

## Abstract

*Mirabilis himalaica* (Edgew.) Heimerl is one of the most important genuine medicinal plants in Tibet, in which the special plateau habitat has been associated with its excellent medicinal quality and efficacy. However, the mechanisms by which environmental factors affect biosynthesis of secondary metabolic components remain unclear in this species. In this study, RNA sequencing and iTRAQ (isobaric Tags for Relative and Absolute Quantification) techniques were used to investigate the critical molecular “events” of rotenoid biosynthesis responding to UV-B radiation, a typical plateau ecological factor presented in native environment-grown *M. himalaica* plants. A total of 3641 differentially expressed genes (DEGs) and 106 differentially expressed proteins (DEPs) were identified in *M. himalaica* between UV-B treatment and control check (CK). Comprehensive analysis of protein and transcript data sets resulted in 14 and 7 DEGs from the plant hormone signal transduction and phosphatidylinositol signaling system pathways, respectively, being significantly enriched. The result showed that the plant hormone signal transduction and phosphatidylinositol signaling system might be the key metabolic strategy of UV-B radiation to improve the biosynthesis of rotenoid in *M. himalaica*. At same time, most of the DEGs were associated with auxin and calcium signaling, inferring that they might drive the downstream transmission of these signal transduction pathways. Regarding those pathways, two chalcone synthase enzymes, which play key roles in the biosynthesis of rotenoid that were thought as the representative medicinal component of *M. himalaica*, were significantly upregulated in UV-B radiation. This study provides a theoretical basis for further exploration of the adaptation mechanism of *M. himalaica* to UV-B radiation, and references for cultivation standardization.

## 1. Introduction

*Mirabilis himalaica* (Edgew.) Heimerl (*M. himalaica*) is a genuine medicine of Tibet that has been historically employed in nourishing the kidney, regenerating tissue, for diuresis, and in removing kidney stones, with accounts that could be traced back to more than 1300 years ago [1,2]. However, the wild resources of *M. himalaica* are distributed in a small region, and thus cannot fulfill market demand [3]. Artificial planting has been effectively employed to resolve the shortage of wild resources of *M. himalaica*. Therefore, understanding the relationship between habitat conditions and the growth and quality of this medicinal material is thus essential for standardized cultivation [4,5].

*M. himalaica* grows in hillsides or grasses at an altitude of 2700–3400 m, which are characterized by a plateau dry monsoon climate that features strong ultraviolet radiation, large temperature differences between day and night, and drought. High level of UV-B radiation is a major ecological feature of plateau habitats that largely influence plant growth. Numerous studies have shown that UV-B (280–320 nm) is a biologically effective radiation, and most plants are subject to the stress and generate stress responses upon exposure [6]. For example, numerous studies have shown that enhanced UV-B radiation leads to dwarfing of plants and shortening of internodes [7,8]. However, plants can compensate for the reduced leaf area caused by UV-B radiation by increasing the number and thickness of leaves [9]. Increasing the wax content of the leaf surface is another plant response to UV-B radiation stress [10]. However, for medicinal plants, in addition to influencing growth and development, UV-B radiation acts as a regulatory factor that increases the production and accumulation of pharmaceutically active ingredients [11]. For example, increased UV-B radiation can significantly enhance anthocyanin and carotenoid content in chrysanthemum [12]. UV-B radiation significantly increases adenosine, cordycepin and ergonovine content in *Cordyceps militaris* sporocarp [13]. UV-B radiation can also promote the synthesis of indigo and indirubin in *Folium isatidis* and *Radix isatidis*, thereby improving the quality [14]. Many studies have found that UV-B radiation could significantly promoted the accumulation of hydrogen peroxide in cells, further leading to an increasing of free radical levels and producing oxidative stress [15,16,17,18]. Therefore, the enzyme activities of some representative antioxidant enzymes were frequently used to judge the damage degree and adaptability of plants to UV-B stress, such as catalase (CAT), peroxidase (POD) and superoxide dismutase (SOD) [17,18].

Currently, *M. himalaica* has adapted to high levels of UV-B radiation in plateau habitats. However, details on the adaptation mechanism of *M. himalaica* and its metabolic responses are unclear. Therefore, the present study measured the dynamic changes of the physiological index and the content of rotenoid as a medicinal active ingredient of *M. himalaica* to UV-B radiation. Simultaneously, RNA sequencing and iTRAQ techniques were used to investigate the critical molecular “events” of rotenoid biosynthesis responded to UV-B radiation. This study provides an important scientific basis for interpreting the molecular mechanism underlying metabolism and standardization of *M. himalaica* in plateau habitats.

## 2. Results

### 2.1. The Physiological Response of M. himalaica to Different Dose of UV-B Radiation

**Figure 1:**
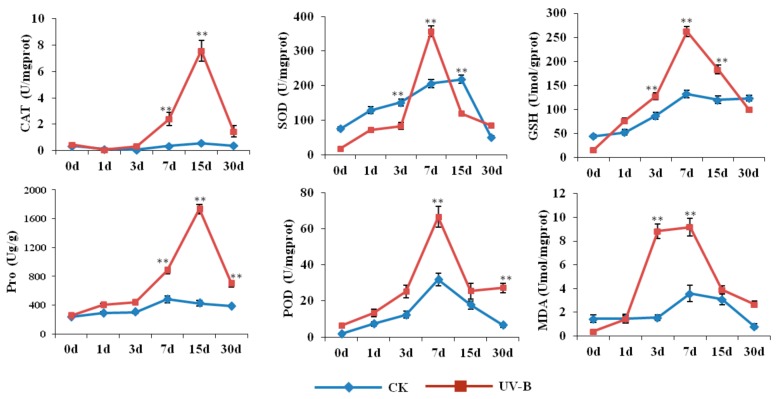
Comparison of physiological indices of the roots of *M. himalaica* from the UV-B radiation and control check (CK) groups. The abscissa is the treatment time for *M. himalaica* from pre-treatment (Day 0) to Day 30 after treatment. The ordinate indicates the different enzyme activities or substance content. CAT: catalase; SOD: superoxide dismutase; GSH: glutathione; Pro: proline; POD: peroxidase; MDA: malondialdehyde. The “**” represented the significant difference level between treatments (*p*-value < 0.01).

The formations of genuine medicinal materials were actually the results of plant interaction with environmental factors. Roots of *M. himalaica* as the traditional medicinal parts were used for clinical treatment after processing. Therefore, the influences of environmental factors on roots of *M. himalaica* directly determined the quality of medicinal materials. To determine the effects of UV-B radiation on *M. himalaica*, dynamic changes of physiological indexes in the roots of *M. himalaica* under CK and UV-B radiation conditions were assessed (Figure 1). The activity of antioxidant enzymes in *M. himalaica* under different UV-B radiation conditions varied from those of the CK group. For example, CAT and POD activities significantly increased after three days of UV-B radiation treatment, and peaked on days 15 and 7, respectively, and then POD came to a new balance period. However, glutathione (GSH) and SOD levels did not significantly change with UV-B radiation until day 7. Malondialdehyde (MDA) content significantly increased after three days of UV-B treatment and then significantly decreased on day 15. In addition, dynamic changes in proline (Pro) content were observed, which coincided with changes in CAT and POD activity. These findings indicate that UV-B irradiation imparts various induction effects on *M. himalaica* after seven days of exposure.

**Figure 2:**
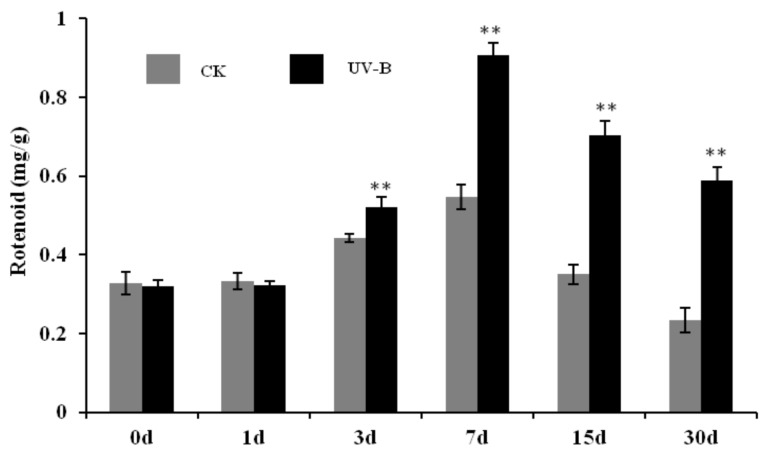
Analysis of rotenoid content in the roots of *M. himalaica* compared UV-B radiation with (CK). The “**” represented the significant difference level between treatments (*p*-value < 0.01).

To further study the effect of UV-B radiation on rotenoid content in *M. himalaica*, the present study also investigated changes in rotenoid content in the roots of *M. himalaica* with UV-B treatment. The results showed that rotenoid content in the roots of *M. himalaica* increased after three days of UV-B radiation compared to that of the CK group. A greater increase in rotenoid content was observed in *M. himalaica* after 7 days of UV-B exposure (Figure 2). During the whole treatment process (from 30 days to 60 days after seedling emergence), rotenoid content in the roots of *M. himalaica* in the CK and UV-B treatment groups ranged from 0.2347 mg/g to 0.5472 mg/g and 0.3199 mg/g to 0.9066 mg/g, respectively. There was a significant difference in rotenoid content between the two groups on day 7, and this trend was maintained in the later stages of treatment. In addition, rotenoid content in different treatments decreased after seven days, which may be due to the dilution effect caused by the rapid growth of *M. himalaica* roots at 40 days after seedling emergence. Therefore, samples subjected to seven days of treatment were selected for transcriptome sequencing and proteomics identification to further explore the molecular and metabolic characteristics of *M. himalaica* responses to UV-B radiation.

### 2.2. Construction and Functional Analysis of the Transcriptome Library for M. himalaica

The transcriptomes of M. himalaica root samples after seven days of UV-B treatment and the CK were sequenced using Illumina Hiseq4000, and each group was assessed in triplicate. A total of 50,820,034, 65,853,560, and 47,847,484 raw reads were obtained from three samples from the CK group, and 58,188,756, 57,886,202, and 43,004,860 raw reads were obtained from three samples from the UV-B treatment group. All clean reads were mixed assembled using Trinity software, and resulting 64,027 unigenes were obtained. Then, the clean reads data from six samples were separately mapped to above 64,027 unigenes. Of which, 59,966, 56,050, and 53,739 unigenes were got from the CK group, and 56,629, 58,837, and 59,494 unigenes were got from UV-B treatment group (Table 1).

**Figure 3:**
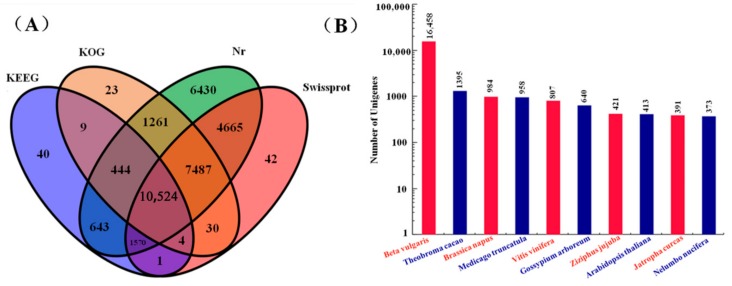
Annotation of genes in various databases and statistics of homologous genes. (**A**) Annotation of *M. himalaica* transcriptome library assembly genes in different databases. (**B**) Statistics on the number of homologous genes between *M. himalaica* and other species.

**Figure 4:**
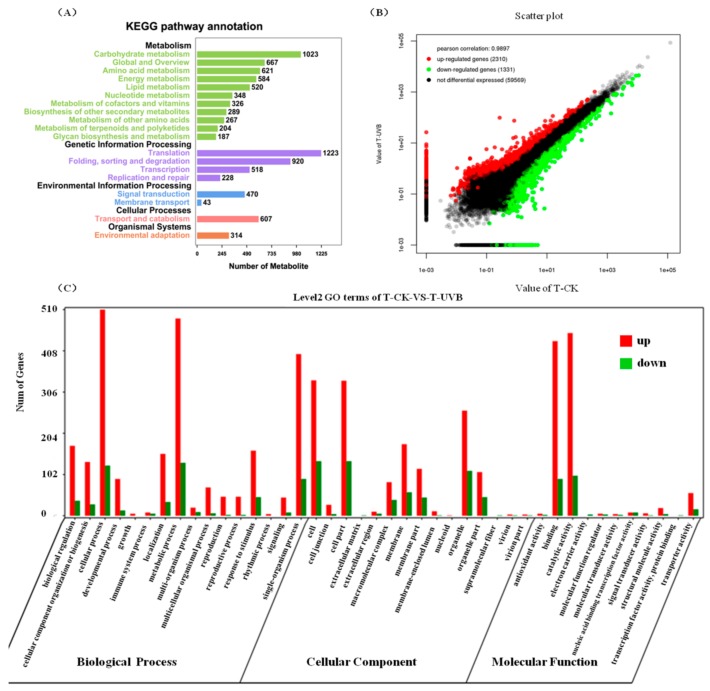
Functional annotations of transcribed conserved domain (CD)-containing sequences and differentially expressed volcano plots between UV-B radiation and CK. (**A**) Kyoto Encyclopedia of Genes and Genomes (KEGG) classification of transcribed CD-containing sequences in *M. himalaica*. (**B**) Differentially expressed volcano plots between UV-B radiation and CK. In this study, the MARS (MA-plot-based method with Random sampling model) model in the DEGseq v1.20.0 package (http://bioconductor.org/packages/release/bioc/html/DEGseq.html) was used to confirm whether there is a difference between different treatments based on the criteria both |fold change| > 2 and *p*-value < 0.05. (**C**) Go functional annotation of different expression unigenes between UV-B radiation and CK.

The *M. himalaica* unigenes were functionally annotated using databases such as Nr (ftp://ftp.ncbi.nlm.nih.gov/blast/db/), KEGG (https://www.genome.jp/kegg/), and KOG (ftp://ftp.ncbi.nih.gov/pub/COG/COG2014/data/). A total of 33,173 unigenes could be functionally annotated to databases, including 33,024 in the Nr database, 24,323 in the Swiss-Prot database (https://www.uniprot.org/), 19,782 in the KOG database, and 13,235 in the KEGG database (Figure 3A). Of these annotated unigenes, 10,524 were annotated in all four databases. Sequence alignment indicated that the species with the highest number of homologous genes in *M. himalaica* was *Beta vulgaris*, which had a total of 16,458 homologous genes (Figure 3B). Based on the KOG database, 19,782 unigenes can be annotated into 25 functional categories. General function prediction (6787), posttranslational modification (4254), and signal transduction mechanisms (3399) are the classifications with the highest numbers of matched genes (Figure S1). Gene Ontology (GO) analysis of *M. himalaica* showed that there were 9352, 8961, and 7010 genes that could be classified under the functional category of biological process, and the top three GO terms were metabolic process, cellular process, and single-organism process. The top three GO terms that were annotated in the functional category of cellular component were cell, cell part and organelle, with is 6838, 6836, and 5292 unigenes, respectively. In terms of molecular function, the genes were mainly distributed to the GO terms of catalytic activity and binding, and the number of assigned genes was 8380 and 7511, respectively (Figure S2). For KEGG pathway analysis, 13,235 sequences were successfully annotated to 19 subclasses that were involved in metabolism or signaling pathways (Figure 4A). Approximately 5036 unigenes were functionally annotated to the metabolism category, and 2889 unigenes were classified under the category of genetic information processing, and 513 unigenes belonged to the environmental information processing category.

### 2.3. Identification of Key Genes in Responding to UV-B Radiation by Transcriptome Analysis

To identify the key response genes to UV-B radiation in *M. himalaica*, the standards of |fold change| > 2, *p*-value < 0.05 were used to identify the differentially expressed genes (DEGs) between treatments, by the MA plot-based method with random sampling model (MARS) model in the DEGseq v1.20.0 package. A total of 3641 DEGs were identified between the UV-B treatment and CK groups, of which 2310 were upregulated and 1331 were downregulated (Figure 4B). GO enrichment analysis showed that the DEGs after UV-B treatment were mainly under the functional categories of cellular process, metabolic process, the single-organism process, biological regulation, and responses to stimuli, with 634, 619, 491, 210, and 207 DEGs, respectively, indicating that these processes play major roles in the responses of *M. himalaica* to UV-B radiation. Catalytic activity (551), binding (523), and transporter activity (72) were the most abundant molecular function categories, indicating that catalysis and transport of substrates are critical in the responses to UV-B radiation. For the functional category of cellular components, the cell and organelle-related components were the largest groups of unigenes. Additionally, the number of upregulated genes was greater than the number of downregulated genes, which coincided with the expression pattern of DEGs between treatments (Figure 4C).

Of the 3641 DEGs, 483 genes had corresponding functional annotations in the KEGG pathway, which included 97 metabolic pathways. The metabolic pathways of plant hormone signal transduction, phosphatidylinositol signaling system, carotenoid biosynthesis, and flavonoid metabolism pathways were significantly enriched between the two treatments. Studies have suggested that these pathways play important roles in the responses of *M. himalaica* to UV-B radiation (Table 2). The DEGs involved in plant hormone signal transduction include 10 auxin signal transduction genes, 1 auxin influx carrier, 5 auxin-responsive protein IAA (*AUX*/*IAA*s), 1 Gretchen Hagen 3 (*GH3*), and 3 small auxin-up RNAs (*SAUR*s). In addition to the downregulated expression of the three SAUR genes, the remaining seven genes involved in auxin signaling were upregulated under UV-B irradiation. Furthermore, two abscisic acid receptors (*PYL*s) involved in abscisic acid signal transduction, one TIFY family gene (*TIFY*) involved in jasmonic acid signal transduction, and one ethylene insensitive 3 (*EIN3*) involved in ethylene signal transduction were upregulated after UV-B radiation treatment. Among the DEGs involved in the phosphatidylinositol signaling system, phosphatidylinositol 4-phosphate 5-kinase (*PIP5K*) and diacylglycerol kinase (*DAGK*) were upregulated with UV-B treatment. Of the five calmodulin genes, four genes were upregulated (Unigene0060974, Unigene0061365, Unigene0053452, and Unigene 0039588), and one gene was downregulated (Unigene0546615). Specifically, three of the calmodulin genes (Unigene0060974, Unigene0061365, and Unigene0053452) were expressed under UV radiation conditions. Changes in plant signal transduction lead to corresponding changes in material metabolism. In this study, changes in secondary metabolism mainly occurred in carotenoid biosynthesis and flavonoid metabolism, which involved four and three DEGs, respectively. The four DEGs in the biosynthetic pathway of carotenoids included one phytoene synthase (*PSY*) and three carotenoid cleavage dioxygenase (*CCD*) and are upregulated under UV-B radiation conditions. The DEGs involved in flavonoid biosynthetic include two chalcone synthase (*CHS*) and one 4-coumarate-CoA ligase (*4CL*) genes, which are upregulated under UV-B irradiation conditions. Interestingly, the rotenoid biosynthetic pathway derived from the downstream branch of the flavonoid biosynthesis pathway. Our previous studies have suggested that the genes involved in rotenoid biosynthetic pathway mainly included phenylalanine ammonia-lyase, cinnamate-4-hydroxylase, *4CL*, chalcone isomerase and *CHS*. However, excepting for *CHS* (Unigene0055255, Unigene0017851) and *4CL* (Unigene0016937), remaining genes on the rotenoid biosynthetic pathway were not significantly differentially expressed the between treatments. In addition, a specific gene (Unigene0003427) that were annotated as ultraviolet-B receptor UVR8 was significantly upregulated for 2.07 folds in UV-B treatment, implying that UVR8 was high-efficiency expressed in the roots of *M. himalaica* under UV-B stimulus. These signal transduction pathways and the differential expression of genes on the metabolic pathway provide important basic information on the mechanisms of adaptation and metabolic responses of *M. himalaica* to UV-B radiation.

### 2.4. Construction of Protein Library for M. himalaica and Functional Analysis

In a parallel analysis, a comparative proteome survey was performed on the UV-B treatment and CK groups using the iTRAQ technique to complement the transcriptome study. In this study, 21,587 peptides were detected by iTRAQ technology, and 3807 proteins were identified. According to the molecular weight, the protein detection results were as follows: 10–20 KDa (480), 20–30 KDa (611), 30–40 KDa (625), 40–50 KDa (531), 50–60 KDa (426), and 60–70 KDa (339). Of the 3807 proteins identified, 2940 proteins were identified in all samples tested. Based on GO annotation information on the 2940 proteins, these proteins are mainly involved in cellular processes (1701), metabolic processes (1986), single-organism processes (1436), cell (1703), cell parts (1702), binding (1476), and catalytic activity (1681) (Figure S3). In KEGG pathway annotation, 1871 proteins were successfully annotated to 120 metabolic or signaling pathway subclasses (Table S1). Functional annotation information of these proteins provides important information for further study of *M. himalaica* adaptation strategies for UV-B radiation at the protein level.

### 2.5. Identification of Key Proteins Related to UV-B Radiation

**Figure 5:**
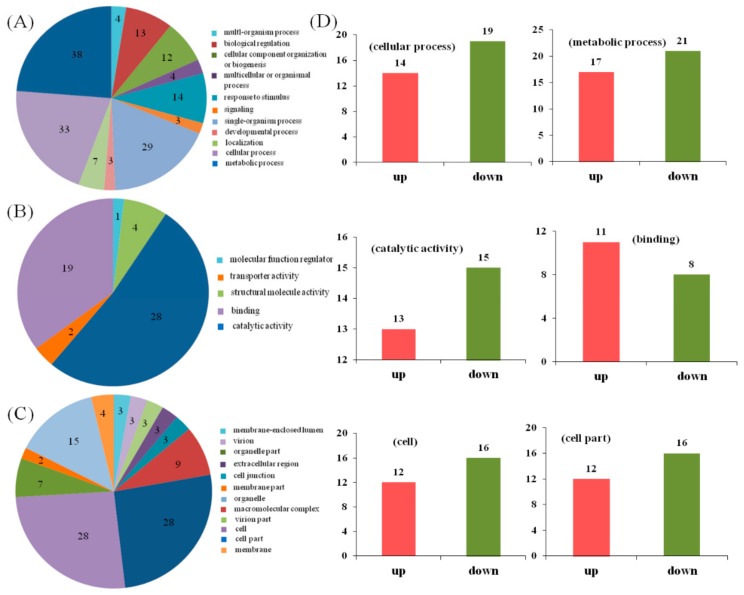
Gene Ontology (GO) function annotation of differentially expressed proteins between different treatments and differential expression analysis of major GO terms. (**A**–**C**) GO function annotation of differentially expressed proteins between different treatments, (**A**) biological process ontology; (**B**) molecular function ontology; (**C**) cellular components Ontology; and (**D**) Differential expression of major GO terms in different ontology between treatments according to the criteria of |Fold change| > 1.2 and *p*-value < 0.05, which is used to determine whether there is a difference in the expression levels of proteins between different treatments.

The differential protein expression pattern in the treatment group was analyzed according to the criteria of |fold change| > 1.2 and *p*-value < 0.05 in this study. The results showed that there were 106 differentially expressed proteins (DEPs) between the UV-B treatment and CK groups, of which 37 were upregulated and 69 were downregulated (Table S2). GO enrichment analysis showed that the DEPs in the biological process categories after UV-B treatment consisted of the subcategories of metabolic process (38), cellular process (33), single-organism process (29), response to stimulus (14), and biological regulation (13). Catalytic activity (28) and binding (19) were the most highly enriched functional categories. For the functional category of cellular components, the subcategories of cell and organelle-related components were most high enrichments with DEPs (Figure 5A–C). The number of upregulated proteins in these GO terms was significantly lower than the number of downregulated proteins, which coincides with the overall expression pattern of DEPs (Figure 5D).

### 2.6. Combined Analysis of Critical Proteins and Genes Responed to UV-B Radiation

Of the 106 identified DEPs, 24 had corresponding transcripts in the RNA-seq data. Approximately 23 proteins had similar protein abundance and transcriptional expression levels between the two treatments, 19 proteins were downregulated, and 4 proteins were upregulated under UV-B treatment (Table S3). However, contrasting levels were observed for one gene, glutamate dehydrogenase, which exhibited upregulated mRNA expression but downregulated protein abundance. Although some differences in gene transcription and protein levels were observed, our findings provided an important data basis for revealing the molecular mechanism on *M. himalaica* plants in response to UV-B radiation.

### 2.7. Quantitative Analysis of Genes Related to of M. himalaica Responses to UV-B Radiation

**Figure 6:**
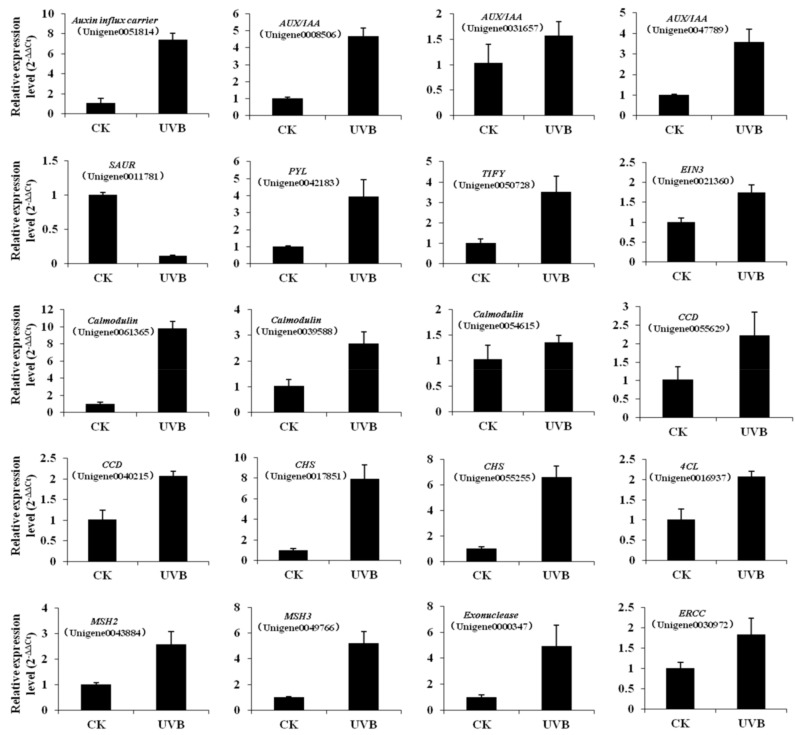
qRT-PCR verification of the key unigenes responding to UV-B radiation.

To further identify the expression patterns of the above genes in different treatments, 20 DEGs, which were identified to involve in key pathways responding to UV-B radiation, were selected to qRT-PCR (Real-time Polymerase Chain Reaction) validation (Figure 6). The results showed that three *AUX*/*IAA* and one auxin influx carrier that are involved in auxin signal transduction were upregulated expression, whereas *SAUR* (Unigene0011781) was downregulated. The three calmodulin genes involved in PI signal transduction were upregulated under UV-B irradiation, whereas calmodulin (Unigene0054615) was downregulated, which may be associated with its functional diversity. The results also indicate the calcium signals play an important role in the responses of the PI signal system to UV-B radiation. Three of the rotenoid biosynthesis genes *CHS* (Unigene0055255, Unigene0017851) and *4CL* (Unigene0016937) were upregulated, indicating that UV-B radiation contributes to rotenoid biosynthesis. In general, except for one calmodulin (Unigene0054615), the results of differential expression analysis of the remaining genes in the treatment group coincided with the findings of transcriptome sequencing, thereby demonstrating the reliability of transcriptome sequencing.

## 3. Discussion

The survival adaptation mechanism and metabolic regulation responses of plants in specific habitats are the basic methods to study plant stress responses. For medicinal plants, the production of the secondary metabolites in a particular habitat is closely related to its pharmacological activity and synthesis of genuine medicines. The high level of UV-B radiation in Tibet induces plants to develop specific external morphologies and internal regulatory mechanisms. *M. himalaica* is a genuine medicine material in Tibet that has adapted to the strong ultraviolet radiation environment of plateaus during evolution and is an ideal material for studying adaptation mechanisms of plants to UV-B radiation. In addition to studies on stress responses of plants to UV-B radiation, recent investigations have focused on the role of UV-B radiation in regulating plant growth and metabolism [11]. RNA-seq and iTRAQ techniques were used in this study to identify differences between the transcriptome and proteome of *M. himalaica* under UV-B and control conditions. Transcriptome analysis has identified 3641 DEGs between the UV-B treatment and CK groups. Of the 106 DEPs identified by iTRAQ, 24 corresponded to gene sequences that were obtained by RNA-seq, thereby enabling the comparison of UV-B response-related differences in specific transcripts or cognate proteins. In the present study, 95.8% (23 out of 24) of the protein and transcript pairs decreased or increased with UV-B exposure. Many genes show consistency at the transcriptional and protein levels. The integrated transcriptome and proteomics data highlight a set of metabolic changes that may be involved in the response and adaptation of *M. himalaica* to UV-B radiation.

The process that plants recognized the external environment factors commonly included three key steps, signal perception, transmitting and cellular reaction. As one of important environment factors, the response of plants to UV-B radiation must contain similar molecular process. Rizzinidn et al. firstly confirmed that UVR8 was UV-B photoreceptor [19]. Thereafter, many studies have shown that the responses of plants to UV-B radiation were mainly mediated by UVR8 [20,21,22]. Among studies for effects of UV-B radiation on plants, Hideg et al thought that different doses of UV-B radiation could increase the level of the reactive oxygen species of plant cells [20]. In this study, some antioxidant physiological indicators of *M. himalaica* were significantly increased in UV-B treated plants (Figure 1), suggested that UV-B treatment promoted accumulation of reactive oxygen species in plants. In addition, a few of studies have shown that UVR8 in cells was not fully determined by light and UV-B radiation [23]. For example, UVR8s were found in the roots of *Arabidopsis* and carrots, which participated in root growth and secondary metabolism [24,25]. In this study, the UVR8 (Unigene0003427) in the roots of *M. himalaica* was significantly upregulated in UV-B radiation compare with CK plants. At same time, the contents of rotenoid as medicinal active compound of *M. himalaica,* were significantly increased under UV-B radiation (Figure 2). These results suggested UVR8 and downstream signaling pathways might affect the adaptation of *M. himalaica* to UV-B radiation and are closely related to the regulation of secondary metabolite biosynthesis.

Phosphatidylinositol (PI) is an important component of the cell membrane and plays an important role in the transmission of environmental signals inside and outside the cell [26,27]. To date, studies on the structure and function of key enzymes involved in the plant PI signaling pathway, such as phospholipase and phosphotidylinositol kinase (*PIK*) [28], have improved our understanding of this signaling pathway. The PI signal system plays an important role in the early growth and development of plants and the signal transduction process of environmental factors in response to the activation of calcium ions by inositol trisphosphate 3 (*IP3*) and the activation of protein kinase C (*PKC*) by diacylglycerol (*DAG*) [29]. Furthermore, that study determined that the key enzymes in the PI signal transduction system of plants are activated by stress. Their expression levels and the phospholipid metabolism activity significantly increased [30]. Here, we found that seven DEGs are involved in PI signaling, and one phosphatidylinositol 4-phosphate 5-kinase and one diacylglycerol kinase were upregulated under UV-B radiation. In addition, the five calmodulin DEGs were identified (four were upregulated and one was downregulated). Three calmodulins were specifically expressed under UV-B radiation conditions. However, the DEG belonging to *PKC* was not detected in this study, and we speculated that the PI signaling system in *M. himalaica* mainly promotes calcium ion-activated release by *IP3*, which further regulates the activity and its associated signal transduction pathways of calcium ion or calmodulin-dependent enzymes responsive to UV-B radiation. In addition to the direct effects, some studies have found that PI signaling systems can indirectly respond to plant growth and development processes and environmental factors by regulating hormones [27]. For example, the PI signaling system plays an important role in regulating the synthesis and signal transduction of abscisic acid (ABA) [31], IAA [32,33] and JA [34].

Plant hormones play an important role in plants responding to specific external environments. In particular, many stress-responsive hormones regulate plant growth and material metabolism with the synergistic or antagonistic effects under stress conditions. Auxin is the first plant hormone found in plants, which participates in almost all plant development processes, and plays an important role in UV-B induced plant domestication and stress response [35]. Most early auxin response genes are classified into three families: *AUX*/*IAAs*, *GH3s*, and *SAURs* [36]. Many studies have shown that *AUX*/*IAAs* and *GH3s* are the two most important negative regulators of auxin response [37,38,39,40]. Usually *AUX*/*IAAs* interact with auxin-responsive promoter elements to form heterodimers to inhibit auxin signal transduction, while *GH3s* negatively regulate shoot, hypocotyl cell elongation and lateral root formation [39,40]. However, based on the acid-growth and gene activation hypotheses, SAURs are thought to mediate cell expansion [41]. The five *AUX*/*IAAs* and *CH3s* (unigene0059280) identified in this study were upregulated under UV-B radiation conditions, whereas three SAURs were downregulated under UV-B radiation conditions. Based on the function of three proteins and their expression trends, we hypothesized that UV-B radiation significantly attenuated the induction effect of auxin in the roots of *M. himalaica*. In addition, many studies have shown that UV-B radiation can induce the synthesis of abscisic acid [42,43], jasmonic acid [44,45] and ethylene [46] in plants, which further promote a series of downstream stress responses in plants. Genes involved in the signal transduction of abscisic acid (unigene0054135 and unigene0042183), jasmonic acid (unigene0050728) and ethylene (unigene0021360) were found upregulated under UV-B radiation conditions in this study. The response genes involved in abscisic acid were identified as *PYL*s. Studies have shown that *PYL*s act as receptors for abscisic acid to inactivate type 2C protein phosphatases in response to ABA signal transduction [47]. Ten of the DEGs involved in plant hormone signal transduction were identified to be related to auxin, abscisic acid, ethylene, and ethylene signal transduction (Table 2) in this study. Based on the function of these genes and their expression trends, it has been suggested that *M. himalaica* is mediated by the regulation of auxin and abscisic acid with the phytohormone signal transduction pathway of jasmonic acid and ethylene signal transduction to respond to UV-B radiation. The results of this study are consistent with the conclusions of Vanhaelewyn et al., namely, that the effects of UV-B on phytohormone regulation generally include inhibition of auxin and enhancement of defensive hormone by environmental stress [35].

Plants produce UV-absorbing substances by secondary metabolism in response to UV-B radiation, which is an effective defense mechanism [48,49,50]. In general, plants synthesize phenolic compounds, olefinic compounds, and carotenoids [51,52], in which phenolic compounds and olefinic compounds are the main UV-absorbing substances [53]. Additionally, these substances are also active ingredients of various medicinal plants such as tanshinone in *Salvia miltiorrhiza*, and flavonoids and total phenol in *Arborvitae*. Medicinal plants not only have all the functions of plants, but their secondary metabolites (medicinal active ingredients) also provide important medical and health effects for human health [54]. Plant secondary metabolites are more reflective of the correlation and adaptability between plants and the environment than primary metabolites [55]. Studies have shown that UV-B radiation has an important influence on the accumulation of active constituents of medicinal plants. For example, Chen found that UV-B radiation can promote the synthesis of indigo and indirubin in *Radix isatidis*, which effectively improve the quality of *Radix isatidis* [14]. Zhou et al. reported that enhancement of UV-B radiation can significantly affect the production of secondary metabolism in *Salvia miltiorrhiza* [56]. Zhou et al. reported that UV-B radiation significantly affected the biosynthesis of alkaloids in *Pinellia ternata*, among which low-intensive UV-B radiation is not beneficial to the synthesis of Pinellia alkaloids, whereas high-intensity UV-B radiation is beneficial to the alkaloid synthesis of *P. ternata* [57]. In this study, DEGs involved in secondary metabolism pathways were mainly associated with flavonoid metabolism and carotenoid biosynthesis pathway. Among them, four DEGs involved carotenoid biosynthesis, and three DEGs involved flavonoid metabolism, which were all upregulated under UV-B radiation (Table 2). It is possible that the metabolic activities of *M. himalaica* are significantly enhanced under the UV-B radiation, and *M. himalaica* adapts to UV-B radiation by increasing the ultraviolet-absorbing substance. Rotenoid, an important medicinal active ingredient in *M. himalaica*, is one of the downstream products of flavonoid metabolism [58,59]. Previous studies on rotenoid have been focused extensively on its anti-cancer activity and the treatment of leukemia [60,61,62]. However, there is no study of the relationship between rotenoid biosynthesis and environmental conditions in *M. himalaica*. Here, we found three DEGs involved in flavonoid metabolism include two chalcone synthase (*CHS*) and one 4-coumarate-CoA ligase (*4CL*) (Table 2). The protein encoded by 1 *CHS* gene was also differentially expressed between the two treatments in the comparative analysis of proteomic data. The protein encoded by one *CHS* gene was upregulated under UV-B radiation conditions, which is consistent with the result of the gene’s expression of transcription levels. All the results suggest that UV-B radiation improves the biosynthesis of rotenoid, a medicinal active ingredient of *M. himalaica*, which is mainly achieved by a series of regulatory activities induced by the PI signaling system and hormone signaling to promote the up-regulation of *CHS*. This result is consistent with previous studies that showed *CHS* is a key gene for rotenoid biosynthesis in *M. himalaica* [58,59].

**Figure 7:**
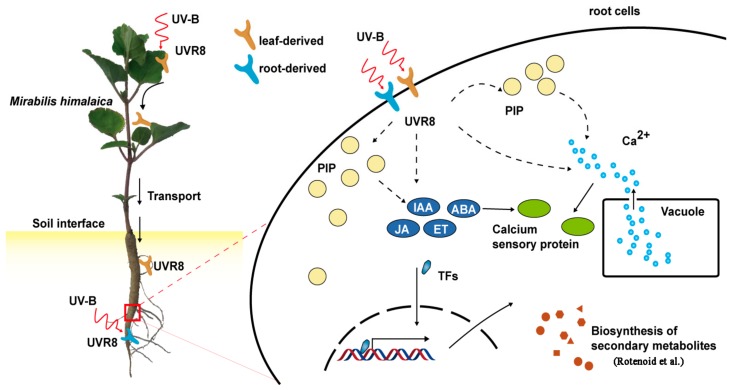
Key molecular pathways for *M. himalaica* response to UV-B radiation.

Through a comprehensive analysis of transcriptome and proteomics, this study shown that the phosphatidylinositol and plant hormone signaling including auxin, abscisic acid, jasmonic acid and calcium signals, might involv in regulating of rotenoid biosynthesis in *M. himalaica* by UVR8 proteins generated under UV-B radiation. Although the complex relationship among these molecular events was currently not clear enough in *M. himalaica*, some critical molecular events in *M. himalaica*, which related to rotenoid biosynthesis regulation caused by UV-B radiation, could be primarily proposed based on results in this study (Figure 7). This study provided a improtant theoretical basis and references for the standardized cultivation and rotenoid biosynthesis regulation of *M. himalaica*.

## 4. Materials and Methods

### 4.1. Test Setup and Collection of Plant Materials

**Figure 8:**
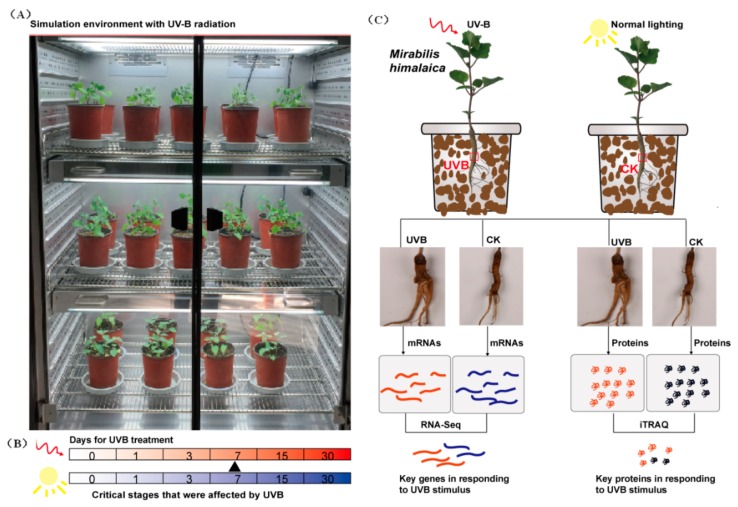
Experimental processing settings and operational procedures. (**A**) Artificial climate chamber used for the experiment; (**B**) Experimental processing settings, the number indicates the days of UV-B radiation; and (**C**) Samples collection and experimental procedures.

The present study employed artificial climate boxes (Binder, Germany) at the Tibetan Medicine Science and Technology Demonstration Park of the Tibet Agriculture and Animal Husbandry Institute in pot experiments involving *M. himalaica* subjected to artificial controlled conditions. *M. himalaica* seeds were seeded (*N* = 8 per pot) on 28 May 2016. The seedlings were sampled seven days after sprouting, and three plants exhibiting similar growth potential were selected for each subsequent test. Thirty days after the emergence of seedlings, the pots were individually transferred to two artificial climate chambers representing two groups, namely, control (CK) and UV-B radiation treatment. Each treatment was performed in triplicate. The potted plants were placed in an artificial climate chamber for one day, and UV-B radiation treatment was initiated. The environmental conditions of the CK group included a temperature of 22 °C during the day (9:00 A.M. to 9:00 P.M.) and 12 °C at night (9:00 P.M. to 9:00 A.M.), and 12 h of light at 15,000 Lux. The soil relative water content was maintained at 60–70%. The air humidity was 60%. UV-B treatment was set on the basis of CK, i.e., Dose of UV-B radiation increased daily at a rate of 100 μW/cm^2^ (equivalent to the average intensity of UV-B radiation contained in nature lighting on daytime in Linzhi City, China, from June to July), and the treatment lasted for 10 h (10:00 A.M.–10:00 P.M.). Radix samples of *M. himalaica* under CK and UV-B treatment conditions were collected at 0 (before treatment), 1, 3, 7, 15, and 30 days. All samples were wrapped in foil and immediately placed in liquid nitrogen for cryopreservation and used in the detection of various physiological indexes. Based on the results, samples from critical treatment periods were selected for transcriptome and proteome analyses (Figure 8).

### 4.2. Physiological Index Determination

Sample tissue was ground with nine times the volume of physiological saline, and then the mixture was centrifuged at 3500 rpm for 10 min. The supernatant was collected and stocked in the refrigerator at 4 °C until use. Subsequently, the supernatant and the control were further processed using a corresponding kit (Nanjing Jiancheng Bioengineering Institute production, Nanjing, China), following the manufacturer’s recommendations. Finally, absorbance measurements were conducted using a fully automated biochemical analyzer (Rayto Production, Shenzhen, China), and the activities of antioxidant system-related SOD, peroxidase (POD), and catalase (CAT) and the contents of glutathione (GSH), malondialdehyde (MDA), and proline (Pro) in the samples were calculated as described elsewhere (http://elder.njjcbio.com/index_en.php). Rotenoid content in the roots of *M. himalaica* was determined as described elsewhere [63].

### 4.3. Library Preparation and Illumina Hiseq4000 Sequencing

The total RNA was isolated using an EASYspin Plus Plant RNA isolation kit (Aidlab, Beijing, China). A 2100 Bioanalyzer was used to perform the total RNA quality control (QC) check. The qualified RNA samples were then digested by DNaseI (Takara, Tokyo, Japan) at 37 °C for 30 min and purified using Dynabeads^®^ Oligo (dT) 25 (Life, Carlsbad, CA, USA). Afterward, 100 ng of purified mRNA was used to establish a library using a NEBNext^®^ Ultra™ RNA Library Prep Kit for Illumina (NEB, Beverly, MA, USA). First, random primers and First Strand Synthesis Reaction Buffer (5×) was added to disrupt the mRNA, after which the mRNA was incubated at 94 °C for 15 min. The sample was chilled on ice immediately afterward. The forward strand of the cDNA was synthesized by adding murine RNase inhibitor and ProtoScript II Reverse Transcriptase to the sample, while the reverse strand of the cDNA was synthesized by adding Second Strand Synthesis Reaction Buffer (10×) and Second Strand Synthesis Enzyme Mix to the sample. The samples were then purified by adding 1.8× the volume of AMPure XP Beads (Agencourt, Beverly, MA, USA), after which the bases were repaired, and uracil was removed. Afterward, 0.6 times the volume of AMPure XP Beads was added to the sample; the mixture was thoroughly mixed and then left to stand for 5 min, after which it was placed on a magnetic rack for 5 min. The supernatant was then removed, and 0.25× AMPure XP Beads was added for purification. Ligated cDNA that ranged from 300–500 bp in size was subsequently retrieved. The samples were then amplified by 15 rounds of PCR and purified using an equivalent volume of AMPure XP Beads. The library was then retrieved and quantified using Qubit, 2% agarose electrophoresis, and a High-Sensitivity DNA chip to ensure the quality of the library. Finally, 10 ng of the library was used to generate clusters with cBot using a TruSeq PE Cluster Kit (Illumina, San Diego, CA, USA) followed by two-way sequencing on an Illumina HiSeq™ 4000/MiSeq™. All the data have been registered in the NCBI Sequence Read Archive (SRA) database (https://trace.ncbi.nlm.nih.gov/Traces/sra/) under GenBank accession number SRP155676 (SAMN09736012, SAMN09736013, SAMN09736014, SAMN09736015, SAMN09736016, SAMN09736017). Three biological replicates were performed.

### 4.4. De Novo Assembly and Annotation

The raw paired-end reads were trimmed using Trimmomatic version 0.32, and QC was performed on the reads using FastQC version 0.10.0; the default parameters were used. The clean data from the libraries were then used for RNA de novo assembly with Trinity version 2.06. All the assembled transcripts were queried against the NCBI protein non-redundant (Nr), Swiss-Prot, and KEGG databases using BLASTX to identify the proteins that had the highest sequence similarity with the given transcripts to retrieve their function annotations. A typical cut-off E-value of less than 1.0 × 10^−5^ was used. InterProScan version 4.8 was used to obtain GO annotations of unique assembled transcripts for describing biological processes, molecular functions, and cellular compounds. Metabolic pathway analysis was performed using the KEGG database.

### 4.5. Differential Expression Analysis and Candidate Genes Identification

To identify DEGs between two different samples, we calculated the expression level of each transcript in accordance with the fragments per kilobase of exon per million mapped reads (FPKM) method. SAMtools version 0.1.19 was used to quantify gene and isoform abundances, and DEGseq version 1.20.0 was used for differential expression analysis. In addition, functional enrichment analysis, which included the use of the GO and KEGG databases, was performed to identify which DEGs were significantly enriched in GO terms and metabolic pathways at a Bonferroni-corrected *p*-value ≤ 0.05 compared with those DEGs in the whole-transcriptome background. The GO functional enrichment and KEGG pathway analysis was carried out using GOseq and KOBAS software.

### 4.6. qRT-PCR Analysis

Total RNA (1 μg) from each sample was used to synthesize cDNA using a PrimeScript RT reagent kit (Takara, Tokyo, Japan). Quantitative real-time PCR (qRT-PCR) was conducted using SYBR premix Ex Taq (Takara, Tokyo, Japan). The analyses were carried out in accordance with the procedures described by Gu et al. [58] but with an annealing temperature of 62 °C. The 18S gene was selected as an internal control for normalizing the expression of the genes detected; the expression levels of this gene were more stable than those of the transcripts among the samples [58]. The data were normalized based on the 18S rRNA threshold cycle (Ct) value. The root samples were used as controls, and their normalized Ct values were set to 1. The relative expression of stem and leaf genes was calculated using the 2^−ΔΔ*C*t^ method. The specific primer pairs used are listed in Table S4. Three biological replicates were performed.

### 4.7. Protein Extraction, Digestion and iTRAQ/TMT Labeling

Total proteins were extracted using the cold acetone method. Samples were ground to power in liquid nitrogen, then dissolved in 2 mL lysis buffer (8 M urea, 2% SDS, 1× Protease Inhibitor Cocktail (Roche Ltd. Basel, Switzerland)), followed by sonication on ice for 30 min and centrifugation at 13,000 rpm for 30 min at 4 °C. The supernatant was transferred to a fresh tube. For each sample, proteins were precipitated with ice-cold acetone at −20 °C overnight. The precipitations were cleaned with acetone three times and re- dissolved in 8 M Urea by sonication on ice. Protein quality was examined with SDS-PAGE.

BCA protein assay was used to determine the protein concentration of the supernatant. 100 μg protein per condition was transferred into a new tube and adjusted to a final volume of 100 μL with 8 M Urea. 11 μL of 1 M DTT (DL-Dithiothreitol) was added and samples were incubated at 37 °C for 1 h. Then 120 μL of the 55 mM iodoacetamide was added to the sample and incubated for 20 min protected from light at room temperature. For each sample, proteins were precipitated with ice-cold acetone, then re-dissolved in 100 μL TEAB. Proteins were then tryptic digested with sequence-grade modified trypsin (Promega, Madison, WI, USA) at 37 °C overnight. The resultant peptide mixture was labeled with iTRAQ/TMT tags. The labeled samples were combined and dried in vacuum.

### 4.8. Strong Cation Exchange (SCX) Fractionation and Liquid Chromatography–Tandem Mass Spectrometry (LC-MS/MS) Analysis

The combined labeled samples were subjected to the SCX fractionation column connected with a high-performance liquid chromatography (HPLC) system. The peptide mixture was re-dissolved in the buffer A (buffer A: 20 mM ammonium formate in water, pH 10.0, adjusted with ammonium hydroxide), and then fractionated by high pH separation using Ultimate 3000 system (Thermo Fisher Scientific, Waltham, MA, USA) connected to a reverse phase column (XBridge C18 column, 4.6 mm × 250 mm, 5 μm, (Waters Corporation, San Diego, CA, USA). High pH separation was performed using a linear gradient starting from 5% B to 45% B in 40 min (B: 20 mM ammonium formate in 80% ACN, pH 10.0, adjusted with ammonium hydroxide). The column was re-equilibrated at initial conditions for 15 min. The column flow rate was maintained at 1 mL/min and column temperature was maintained at 30 °C Twelve fractions were collected; each fraction was dried in a vacuum concentrator for the next step.

Peptide fractions were resuspended with 30 μL solvent C respectively (C: water with 0.1% formic acid; D: ACN with 0.1% formic acid), separated by nanoLC and analyzed by online electrospray tandem mass spectrometry. The experiments were performed on an Easy-nLC 1000 system (Thermo Fisher Scientific, Waltham, MA, USA) connected to an Orbitrap Fusion Tribrid mass spectrometer (Thermo Fisher Scientific, Waltham, MA, USA) equipped with an online nano-electrospray ion source. 10 μL peptide sample was loaded onto the trap column (Thermo Scientific Acclaim PepMap C18, 100 μm × 2 cm), with a flow of 10 μL/min for 3 min and subsequently separated on the analytical column (Acclaim PepMap C18, 75 μm × 15 cm) with a linear gradient, from 3% D to 32% D in 120 min. The column was re-equilibrated at initial conditions for 10 min. The column flow rate was maintained at 300 nL/min. The electrospray voltage of 2 kV versus the inlet of the mass spectrometer was used.

The fusion mass spectrometer was operated in the data-dependent mode to switch automatically between MS and MS/MS acquisition. Survey full-scan MS spectra (*m*/*z* 350–1550) were acquired with a mass resolution of 120 K, followed by sequential high energy collisional dissociation (HCD) MS/MS scans with a resolution of 30 K. The isolation window was set as 1.6 Da. The AGC (Automatic Generation Control) target was set as 400,000. MS/MS fixed first mass was set at 110. In all cases, one microscan was recorded using dynamic exclusion of 45 s.

### 4.9. Database Search

The mass spectrometry data were transformed into MGF files with Proteome Discovery 1.2 (Thermo, Pittsburgh, PA, USA) and analyzed using Mascot search engine (Matrix Science, London, UK; version 2.3.2). Mascot database was set up for protein identification using *M. himalaica* reference transcriptome. Mascot was searched with a fragment ion mass tolerance of 0.050 Da and a parent ion tolerance of 10.0 PPM.

### 4.10. Protein Functional Annotation and Analysis

Proteins were annotated against GO, KEGG and KOG database to obtain their functions. The Mascot search results were averaged using medians and quantified. Differentially expressed proteins were identified based on both fold change >1.2 or <0.83 and *p*-value < 0.05 [64,65,66].

## 5. Conclusions

The molecular adaptation characteristics of Tibetan medicinal material *M. himalaica* to UV-B radiation were studied through transcriptomics and proteomics analyses. A total of 3641 DEGs and 106 DEPs were identified in the UV-B treatment and normal growth plants using transcriptome and proteomics analyses, respectively. Furthermore, the key metabolic pathways and molecular characteristics of *M. himalaica* in response to UV-B radiation were systematically described. It is proposed that the plant hormonal and phosphatidylinositol signal included auxin, abscisic acid and calcium signaling, might be the core driving forces that regulated the biosynthesis of rotenoid in *M. himalaica* mediated by UV-B radiation. Meanwhile, UV-B radiation was found to have a significant positive effect on the biosynthesis of the medicinal active substance, rotenoid, of *M. himalaica*, and *CHS* played an important role in the pathway. In addition, the hypothesis that improves the biosynthesis of rotenoid based on induction of phosphatidylinositol and the plant hormone signaling systems mediated exogenous UV-B stimulus in *M. himalaica* was tentatively suggested. These results provide an important theoretical basis and references for the standardized cultivation of *M. himalaica*, as well as basic information-facilitated rotenoid biosynthesis regulation.

## Figures and Tables

**Table 1:** Statistics of different samples reads data production.

Sample	CK1	CK2	CK3	UV-B1	UV-B2	UV-B3
Raw reads	50,820,034	65,853,560	47,847,484	58,188,756	57,886,202	43,004,860
Clean reads	49,620,668	64,102,640	46,591,316	56,777,882	56,443,466	42,114,956
Average length (bp)	150	150	150	150	150	150
Raw data	7.62 G	9.88 G	7.18 G	8.73 G	8.69 G	6.45 G
Clean data	7.18 G	9.23 G	6.70 G	8.19 G	8.15 G	6.20 G
Q20	97.42%	97.21%	97.22%	97.33%	97.33%	98.31%
GC content	43.37%	45.50%	45.94%	45.25%	44.44%	43.50%
Unigene number	59,966	56,050	53,739	56,629	58,837	59,494

**Table 2:** A list of some important genes that are differentially expressed between UV-B and CK, and are involved in plant hormone signal transduction, phosphatidylinositol signaling system, flavonoid biosynthesis and carotenoid biosynthesis.

Gene ID	log_2_ratio (UV-B/CK)	Description
**Plant hormone signal transduction**		
Unigene0051814	1.35	*auxin influx carrier*
Unigene0008506	2.66	*AUX/IAA*
Unigene0031657	1.83	*AUX/IAA*
Unigene0053509	1.76	*AUX/IAA*
Unigene0047789	1.43	*AUX/IAA*
Unigene0040950	1.40	*AUX/IAA*
Unigene0059280	1.77	*GH3*
Unigene0004906	−3.29	*SAUR*
Unigene0061366	−1.63	*SAUR*
Unigene0011781	−1.16	*SAUR*
Unigene0054135	2.19	*PYL*
Unigene0042183	1.76	*PYL*
Unigene0050728	1.80	*TIFY*
Unigene0021360	1.29	*EIN3*
**Phosphatidylinositol signaling system**		
Unigene0045244	4.75	*PIP5K*
Unigene0023588	3.20	*DAGK*
Unigene0060974	11.9	*Calmodulin*
Unigene0061365	11.74	*Calmodulin*
Unigene0053452	10.39	*Calmodulin*
Unigene0039588	1.75	*Calmodulin*
Unigene0054615	−1.89	*Calmodulin*
**Flavonoid biosynthesis**		
Unigene0055255	1.89	*CHS*
Unigene0017851	1.52	*CHS*
Unigene0016937	1.55	*4CL*
**Carotenoid biosynthesis**		
Unigene0029438	3.17	*PSY*
Unigene0021932	1.24	*CCD*
Unigene0055629	4.13	*CCD*
Unigene0040215	2.06	*CCD*

## Supplementary Materials

Supplementary materials can be found at http://www.mdpi.com/1422-0067/19/11/3324/s1.

## Abbreviations

DEGs	Differentially
DEPs	Differentially
KEGG	Kyoto
GO	Gene
MARS	MA-plot-based method with random sampling model
FDR	False discovery rate
CHS	Chalcone synthase
SOD	Superoxide dismutase
POD	Peroxidase
CAT	Catalase
GSH	Glutathione
MDA	Malondialdehyde
Pro	Proline
AUX/IAAs	Auxin-responsive protein IAA
GH3	Gretchen Hagen3
SAUR	Small auxin-up RNA
TIFY	TIFY family gene
EIN3	Ethylene insensitive 3
PIP5K	Phosphatidylinositol 4-phosphate 5-kinase
DAGK	Diacylglycerol kinase
PSY	phytoene synthase
CCD	Carotenoid cleavage dioxygenase
4CL	4-Coumarate-CoA ligase
PI	Phosphatidylinositol
IP3	Inositol trisphosphate
PKC	Protein kinase C
