# Supplementary material for: Integrated Analysis of Transcriptomic and Proteomics Data Reveals the Induction Effects of Rotenoid Biosynthesis of Mirabilis himalaica Caused by UV-B Radiation
Source: Int J Mol Sci. 2018 Oct 25;19(11):3324. doi: 10.3390/ijms19113324 (PMC6274831; doi:10.3390/ijms19113324)
Supplement: Supplementary file 1 [file ijms-19-03324-s001.zip › Supplementary Information-Figures.pdf]

Supplementary information

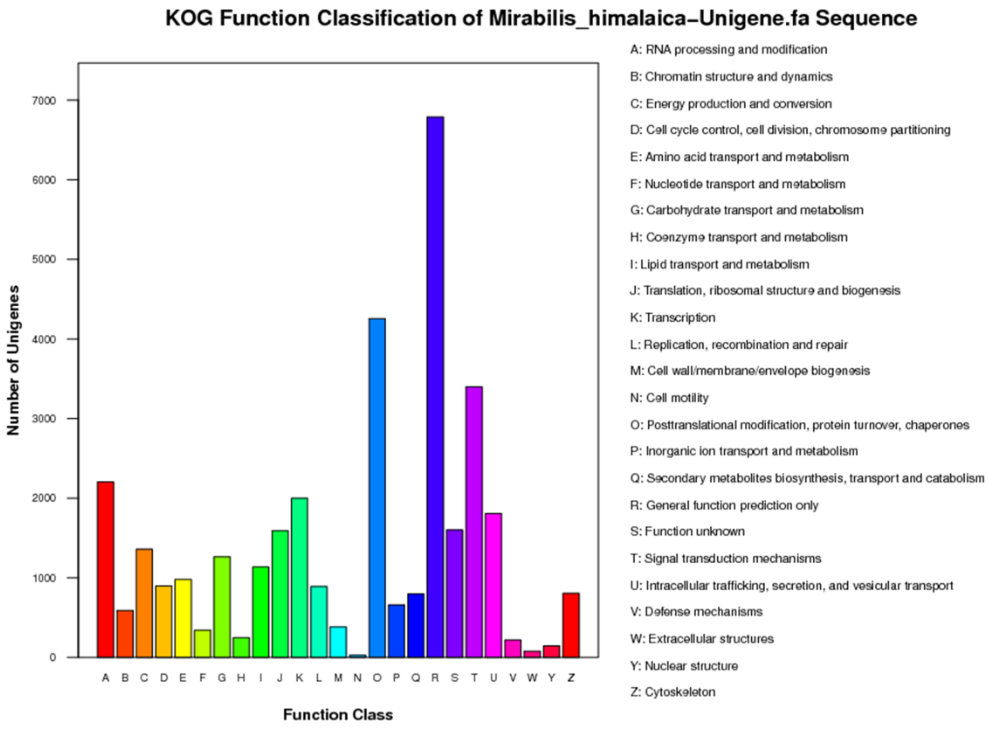

Figure S1. KOG classification of transcribed CD-containing sequences in *M. himalaica.*

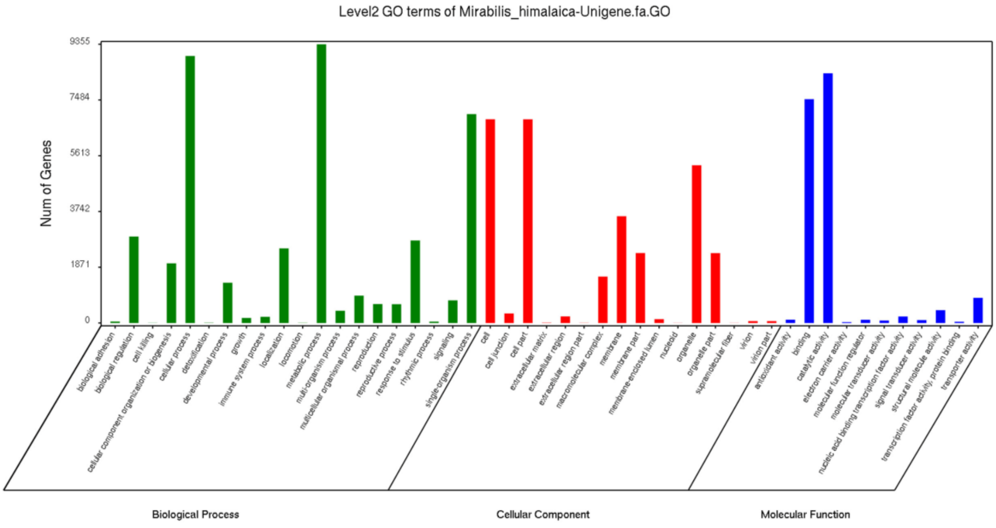

Figure S2. GO classification of transcribed CD-containing sequences in *M. himalaica.*

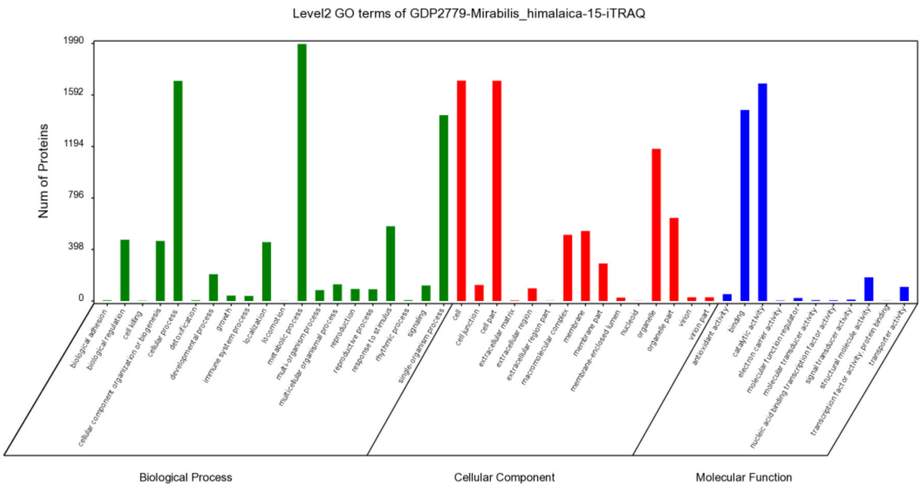

Figure S3. GO classification of *M. himalaica* protein sequence.
